# Visualization of Assembly Intermediates and Budding Vacuoles of Singapore Grouper Iridovirus in Grouper Embryonic Cells

**DOI:** 10.1038/srep18696

**Published:** 2016-01-04

**Authors:** Yang Liu, Bich Ngoc Tran, Fan Wang, Puey Ounjai, Jinlu Wu, Choy L. Hew

**Affiliations:** 1 Mechanobiology Institute, National University of Singapore, Singapore 114543; 2 Department of Biological Sciences; National University of Singapore, Singapore 114543; 3 Department of Biology, Faculty of Science, Mahidol University, 272 Rama VI Rd. Rajdevi, Bangkok, Thailand 10400.

## Abstract

Iridovirid infection is associated with the catastrophic loss in aquaculture industry and the population decline of wild amphibians and reptiles, but none of the iridovirid life cycles have been well explored. Here, we report the detailed visualization of the life cycle of Singapore grouper iridovirus (SGIV) in grouper cells by cryo-electron microscopy (cryoEM) and tomography (ET). EM imaging revealed that SGIV viral particles have an outer capsid layer, and the interaction of this layer with cellular plasma membrane initiates viral entry. Subsequent viral replication leads to formation of a viral assembly site (VAS), where membranous structures emerge as precursors to recruit capsid proteins to form an intermediate, double-shell, crescent-shaped structure, which curves to form icosahedral capsids. Knockdown of the major capsid protein eliminates the formation of viral capsids. As capsid formation progresses, electron-dense materials known to be involved in DNA encapsidation accumulate within the capsid until it is fully occupied. Besides the well-known budding mechanism through the cell periphery, we demonstrate a novel budding process in which viral particles bud into a tubular-like structure within vacuoles. This budding process may denote a new strategy used by SGIV to disseminate viral particles into neighbor cells while evading host immune response.

Iridovirids are large dsDNA icosahedral viruses composed of more than 45 isolates that infect a diverse array of hosts^1^,^2^. Extensive attention has been focused on iridovirids because they are etiologic agents responsible for the dramatic economic loss of cultured fish, frogs, turtles and giant salamanders, as well as causing the decline of the wild population of honey bee colonies, salamanders and reptiles^3^,^4^,^5^,^6^. Scientific interest on iridovirids has also been driven by their evolutionary relationship with other large DNA viruses from the Poxviridae, Asfarviridae, Ascoviridae, Phycodnaviridae, and Mimiviridae families^7^,^8^.

Iridovirid replication has been extensively studied in members from all 5 genera of the family, including invertebrate iridescent virus 6 (IIV6) and *Chilo* iridescent virus (CIV) in genus *Iridovirus*^9^,^10^, mosquito iridescent virus (MIV) in *Chloriridovirus*^4^, lymphocystis disease virus (LDV) in *Lymphocystivirus*^11^,^12^,^13^, red sea bream iridovirus (RSIV) in *Megalocytivirus*^14^, Frog virus 3 (FV3), giant salamander iridovirus (GSIV), epizootic hematopoietic necrosis virus (EHNV), and Singapore grouper iridoivirus (SGIV) in *Ranavirus*^15^,^16^,^17^,^18^.

In brief, iridovirids enter a cell via endocytosis and the viral DNA is transported to the nucleus, where the first stage of viral DNA replication takes place. This is followed by the second stage of DNA replication that occurs in the cytoplasm. The infection subsequently leads to the formation of a morphologically distinct area within the cytoplasm, the viral assembly site (VAS), where the viral proteins and DNA are concentrated for virion assembly. Mature virions accumulate in large paracrystalline arrays or bud from the plasma membrane^1^,^2^,^6^. These studies have used biochemical techniques and imaging by conventional transmission electron microscopy (TEM). However, drawbacks of chemical fixation and low TEM resolution have limited the meaningful interpretation of image data, and the detailed infection mechanism of iridovirids remains largely unclear.

Recently, electron microscopy technology has enabled scientists to make major advances in determining the molecular structures of various macromolecular complexes, including virus particles. The combination of cryofixation and high resolution electron microscopy/tomography (CryoEM/ET) enables analysis of the complex 3D architecture of viral particles and the viral assembly site. Cryofixation involves ultra-rapid cooling of small samples to the temperature of liquid nitrogen, which provides markedly improved structural and biochemical preservation of cells compared to traditional chemical fixation protocols^19^. By avoiding chemical modification of the sample, cells fixed by cryofixation are kept as close as possible to their native states. Therefore, this allows the capture of detailed EM images, or even the presence of unstable and transitory features in biological samples. CryoEM/ET, the most promising platform for studying viral life cycles, has been employed to determine the 3D architecture of viruses, viral intermediates, and virus-induced intracellular structures for gamma herpes virus^20^, human Dengue virus 21, HIV^22^,^23^,^24^, hepatitis virus^20^,^25^ and Cyanophages^26^. However, it has not been applied to study the life cycle of iridovirids.

Singapore grouper iridovirus (SGIV) is a member of the genus *Ranavirus* of Iridoviridae family. It was first isolated in Singapore and has caused catastrophic economic loss in grouper fish farms^27^. To understand its infection mechanism, we have previously determined its molecular compositions including its complete genome, structural proteins and lipids^28^,^29^,^30^. These molecular data build up a solid foundation for a detailed investigation of the viral life cycle and assembly.

This study examined the SGIV life cycle in grouper cells by transmission electron microscopy including cryoEM/ET. We show the detailed process of viral morphogenesis starting from SGIV entry into the cell, viral assembly, and finally egress of viral particles. Knockdown of the expression of SGIV major capsid protein (MCP) significantly impedes the formation of viral intermediates and infectious particles. A novel budding process involving the formation of budding vacuoles is described, which hints at a new immune evasion mechanism used by SGIV.

## Results

### SGIV life cycle

**Figure 1:**
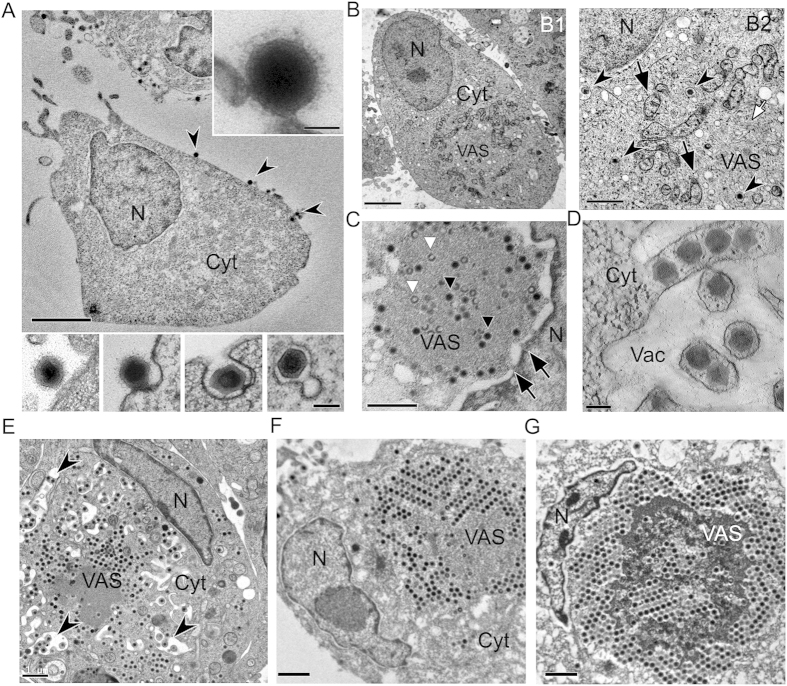
Electron microscopy of high-pressure freezing, freeze substitution of Singapore Grouper Iridovirus (SGIV) infected grouper embryonic cells (GECs) reveals the SGIV life cycle. (**A**) SGIV attaches onto a GEC at 30 min post-infection (arrowheads); scale bar = 1μm. The outermost layer (top inset) around the capsid may initiate the entry process, which takes place through endocytosis (bottom inset, 1 hpi); scale bar = 200 nm. N = nucleus, Cyt = cytoplasm. (**B**) At 1 hpi, the viral assembly site (VAS) starts to form (B1, scale bar = 1μm). Many SGIV particles are still contained in coated vesicles, which is likely to represent SGIV particles in transit (B2, arrowheads, scale bar = 200 nm). Already, some viral intermediates such as membrane filaments can be observed (B2, white arrow). At this time point, mitochondria start to accumulate at the VAS boundary. Some mitochondria appear to have lost their internal structure (B2, black arrows). B2 is an enlarged area of B1. (**C**) At 4–12 hpi, the VAS and nucleus are now interconnected via nuclear pores (arrows), and many partially filled capsids (white triangle) and fully filled capsids (black triangle) are present; scale bar = 1 μm. (**D**,**E**) At 8 hpi, the VAS has expanded dramatically causing the nucleus to deform, and mature virions have accumulated at the edge of the VAS where large vacuoles have formed. Some mature viruses appear to bud into the newly-formed vacuoles containing membrane tubules (**E**, arrowheads; scale bar = 1 μm). The picture D is 220 nm thick tomographic slice showing membrane tubules with a row of mature virions; scale bar = 200 nm. (**F**,**G**) At 48 hpi, the late stage of infection the VAS markedly enlarges and occupies majority of the cell. Mature virions densely accumulate and become packed in the form of paracrystalline arrays inside the cell. The nucleus is pushed to the margin of the cell and now has a highly deformed appearance. The release of mature virions takes place through budding as enveloped virions or through cell lysis as unenveloped virions; scale bars = 1 μm.

In order to study the mechanism of iridovirid infection in their natural host, we used high pressure freezing (HPF) and freeze substitution (FS) to capture the main events of SGIV infection in grouper embryonic cells (GECs). Electron microscopy imaging of the resin-embedded cryofixed specimen revealed various important steps in the SGIV life cycle including entry (Fig. 1A,B), assembly (Fig. 1C), and egress (Fig. 1D–F). At 30 min post infection, SGIV particles were already detected on the plasma membrane of the GECs (Fig. 1A). The enlarged inset (Fig. 1A, upper right corner) clearly shows for the first time that SGIV viral particles have an outer capsid layer. An interaction between the outer capsid layer and the GEC membrane appears to trigger invagination of plasma membrane and viral internalization through endocytosis (Fig. 1A, lower insets). The viral particles contained within the coated vesicles (Fig. 1, B2, arrow head) subsequently traffic to the nucleus (N) where they release their genomic DNA. Unfortunately, we were unable to observe the process of DNA release in our EM specimen, therefore the detail of how viral DNA is released into the nucleus remains unknown. At 1–2 hpi (hours post infection), the viral assembly site (VAS) (Fig. 1, B1) could be observed in the cytoplasm (Cyt). During early VAS formation, mitochondria were recruited to the newly formed VAS (Fig. 1, B1 and B2, black arrows). Some of the mitochondria appeared to be partially disintegrated, probably due to the cytopathic effect caused by the virus. At this stage, the nucleus still occupied a large cellular space. A number of membranous vesicles and tubular membrane structures could be detected inside the early VAS (Fig. 1, B2, white arrow), but no other viral structural components were observed.

At 4–12 hpi, the VAS was completely formed. Various viral intermediates and mature virions could be observed inside the VAS (Fig. 1C, 12 hpi). It should be noted that, depending on section orientation, we observed a direct connection between the VAS and the nucleus via nuclear pores (Fig. 1C, black arrows), potentially suggesting that this pathway is used for trafficking of newly synthesized viral components to the VAS. These connection channels were often observed when the cell (in a resin block) was cut through a certain angle, where the VAS is just adjacent to the nucleus.

At 8 hpi, the VAS expanded in size as mature viral particles accumulate, and this pushed the nucleus to the side of the cell. Interestingly, a novel egression strategy was observed during this stage, in which membrane tubules containing mature virions emerged inside a large cytoplasmic vacuole, forming intracellular tubular networks (Fig. 1D,E, 8 hpi). This unique budding process may give rise to infectious SGIV particles with envelopes derived from the host cell, or represent a route for delivery of newly synthesized viruses directly to neighbor cells via tubular networks.

At 24–48 hpi, the VAS has greatly expanded in size and now occupies the majority of the cytoplasm. This led to further compression of the nucleus at the cell membrane, and it now starts to adopt a deformed morphology. Vast amounts of mature viral particles densely accumulated inside the cell, forming paracrystalline arrays at the boundary of the VAS (Fig. 1F, 24 hpi; Fig. 1G, 48 hpi). These viral particles can leave the cell as enveloped virus via budding out of the cell membrane, or are released as unenveloped particles through cell lysis.

### Capsid assembly and DNA encapsidation

**Figure 2:**
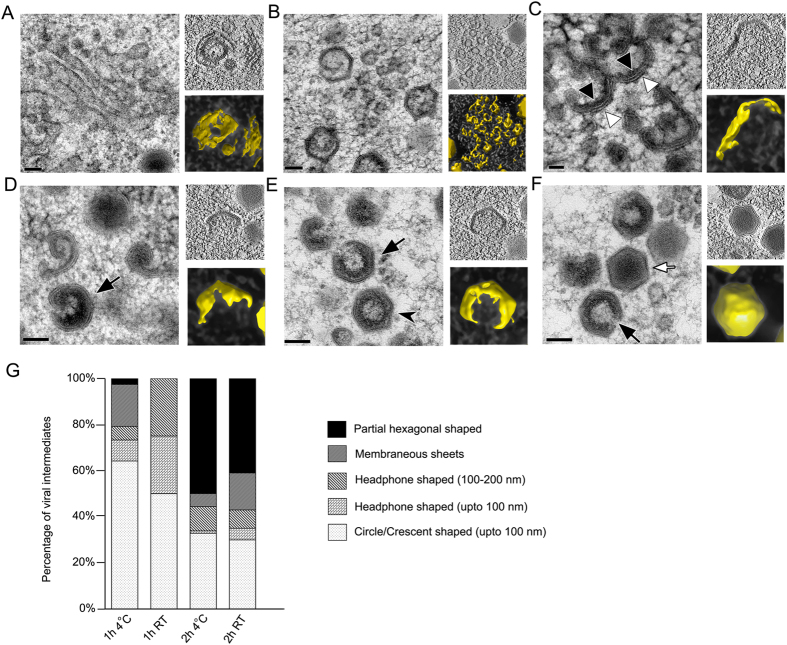
Morphogenesis of SGIV capsids in the VAS. (**A–F**) Main panel, electron micrograph depicting different viral intermediates during capsid assembly; Top right panel, insets of the central slice through a tomogram; Bottom right panel, isosurface rendition of the corresponding viral intermediates (yellow). (**A**,**B**) Membranous vesicles, tubules, and sheets, scale bar = 100 nm. (**C**) Crescent-shaped structures with two shells; scale bar = 50 nm. (**D–F**) Electron dense materials accumulate on the inner layer as the capsid is being filled. As the packing process continues, the two shell crescent-shaped structure curves to form icosahedral capsids with an opening (black arrow). Mature virions (white arrow) with a dense core are eventually formed. No opening was observed on the fully formed mature particles; scale bars = 100 nm. (**G**) The distribution of capsid intermediates observed in infected GECs maintained at 4 °C and 27 °C, at 1 or 2 hpi. Images of the representative intermediates are shown in Fig S1.

To capture the early stages of the capsid assembly, SGIV-infected GECs were maintained at 4 °C to slow the viral replication process. GECs at 1 or 2 hpi were preserved by HPF-FS and subsequently observed via TEM and tomography (Fig. 2A–D). Various membranous structures were observed in the newly formed VAS. These structures, which likely originated from the ER^28^, included large membranous sheets, tubules, and small vesicles (about 30–60 nm in size) (Fig. 2A,B). These membrane structures are believed to constitute a viral inner lipid layer, which later recruits other viral capsid proteins to initiate the formation of a double-shelled structure (Fig. 2C).

During the early stage of capsid formation, crescent shaped intermediates were detected. These intermediates had a double-shell morphology, with each end curving towards the concave side to form the crescent shape (Fig. 2C). These early stage capsid complexes subsequently folded into circular intermediates (Fig. 2D), followed by progressive recruitment of electron dense materials which decorated the inner membrane, resulting in the formation of icosahedral capsids with a small opening on one side (Fig. 2E,F, 24 hpi, GECs grown at RT). Capsids were observed in a partially filled state (Fig. 2, black arrows) as well as fully filled (Fig. 2, white arrows). The insets are tomography images to provide 3D views of these intermediates.

Following our EM/ET imaging of these early intermediates, we carried out quantitative analysis on two different batches of infected GECs to determine the distribution of the viral intermediates. At 1 hpi, the viral intermediates observed were all early stage intermediates, predominantly circle or crescent shaped structures, with some membranous sheets and headphone shaped structures also present. However, by 2 hpi, the percentage of partial hexagonal shaped structures had increased to about 40–50% of the total population (Fig. 2G), supporting our observations, as well as the time scale of the proposed assembly process (Fig. 2A–F). Representative images of the different shaped intermediates are provided in the supplementary data (Fig S1). This distribution was similar irrespective of whether the GECs were grown at 4 ^o^C or 27 ^o^C.

**Figure 3:**
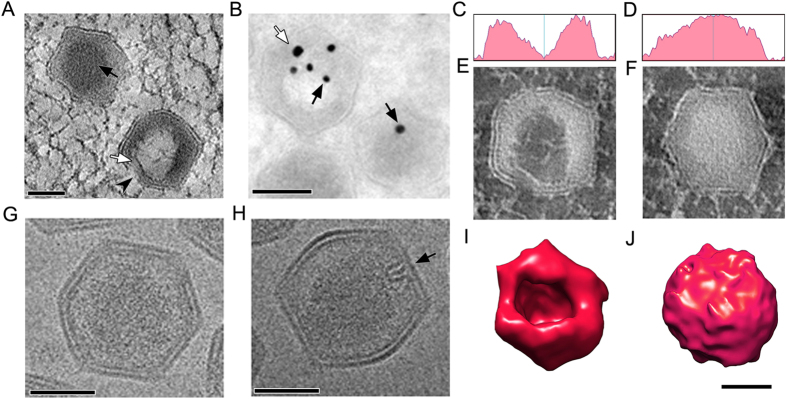
DNA encapsidation. (**A**) A 220 nm tomographic slice of the VAS area revealed partially and fully formed viral particles. In mature virions, electron dense materials are packed to form a compact core inside the viral particle (black arrow), whereas immature virus have a small opening on one side (arrowhead). The electron dense fibrous material forms a lining on the viral capsid inner layer (white arrow); scale bar = 100 nm. (**B**) Immunogold labeling of Tokuyasu section of SGIV-infected cells with anti-viral DNA conjugated with 15 nm gold markers (black arrows) confirmed that the dense materials are viral DNA. Counter-labeling with anti-ORF075 (a DNA core associated protein) conjugated with 25 nm gold markers (white arrow) indicated that ORF075 is located next to the particle opening. Antibodies conjugated with gold can label loosely-packed DNA in the early stage of DNA encapsidation, but the affinity of gold conjugated antibody to label compacted DNA in mature virions is markedly reduced; scale bar = 100 nm. (**C**,**D**) Density plots of the central part of the immature and mature virions, respectively. (**E**,**F**) Inverted contrast imaging of the cross-sections of the corresponding viral particles in (**C**,**D**) demonstrates the density of the packaged genome in SGIV. (**G**,**H**) CryoEM of mature virions revealed the asymmetrical hairpin-shaped complex on one side of the capsid (black arrow). The hairpin structure can only be observed under EM when the sample is at a certain orientation. (**I**,**J**) 3D isosurface rendition of a partially formed particle (**I**) shows a clear opening in comparison with a fully formed, closed mature particle (**J**); scale bar = 100 nm.

Although the capsid opening was larger and easily noticeable in some partially formed viral particles, in many 2D images visualization of the capsid opening was highly ambiguous and orientation-dependent, due to the asymmetrical nature of the viral intermediates. Therefore, we used 3D electron tomographic reconstruction to further verify the existence of the capsid opening in the partially formed virus (Fig. 3). Figure 3A shows a partially formed capsid (white arrow) with the capsid opening (arrow head), and a fully formed capsid (black arrow). Density plots from these two particles (Fig. 3C,D) were created from inverted contrast images (Fig. 3E,F), along with the processed tomography images (Fig. 3I,J). Interestingly, the electron dense material lining the inner layer of the capsid appears to be composed of fibrous material, which is presumably the viral DNA (Fig. 3A). It is thus likely that the capsid opening (Fig. 3A, arrow head) is involved with the viral genome packaging, with subsequent capsid packaging events taking place until the capsid is completely filled. The particles are then sealed to form mature virions. These mature virions can be identified through the presence of highly localized electron dense materials in the centre of the particle (Figs 2F and 3A).

To further investigate whether the electron dense materials were associated with the viral genome, we immuno-labelled viral DNA and a core-associated protein ORF075R with antibody-conjugated gold markers of 15 nm and 25 nm respectively using the Tokuyasu method. The gold particles of 15 nm diameter were abundantly detected in partially-filled capsids but were less abundant in fully filled capsids (Fig. 3B, black arrows). As expected, the core-associated protein ORF075R was also detected with the 25 nm gold marker at a location near the capsid opening (Fig. 3B, white arrow). These data clearly identifies that the electron dense materials are part of DNA in the viral genome. Furthermore, these results may reflect that loosely packaged DNA at the early stage of encapsidation is much more accessible than the highly packaged DNA at the late stage for immune-gold labeling. Interestingly, cryoEM images of purified SGIV particles revealed a distinct feature that looks like a tightly packed loop, located at the middle of one side of the icosahedral structure (Fig. 3H). This feature might either represent the viral sealing marker, which marks the point of the capsid closure, or the portal complex that functions in the ejection of viral genetic material. This unique loop structure was only be observed in a few particles, and appeared to be orientation specific, possibly due to the asymmetric nature of the particles (Fig. 3G,H).

### Role of ORF072 on virus assembly

To analyze the role of SGIV MCP (ORF072) on the viral capsid assembly, GECs that had been transfected with asMO^072^ were infected with SGIV and harvested at 48 hpi. The efficiency of asMO^072^ knockdown on reduction of MCP was assessed by MALDI-TOF/TOF MS/MS. The yield of infectious particles was examined by TCID_50_, and the VAS was visualized using TEM.

**Figure 4:**
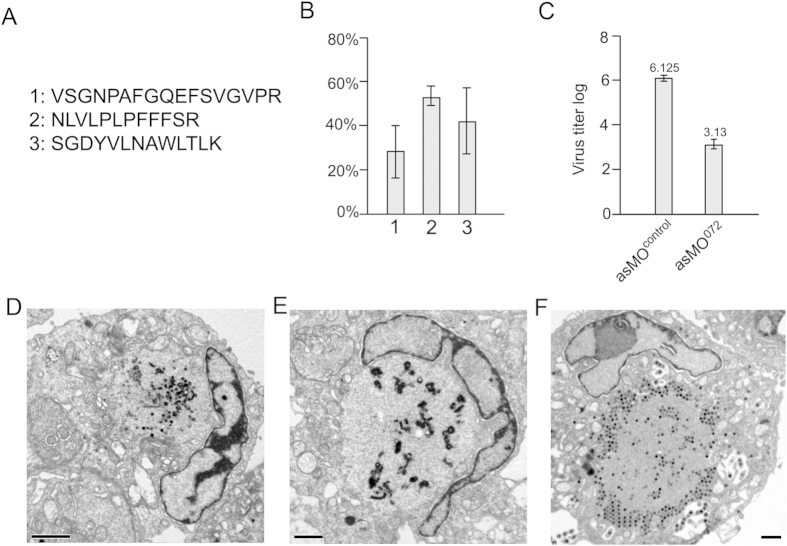
Knockdown of major capsid protein (MCP, ORF072). (**A**) The sequence of the three peptides of MCP detected by mass spectrometry. (**B**) Morpholino-mediated knockdown of MCP resulted in reduced abundance for all three peptides. The comparison is based on the peak areas of identified peptides, calculated using Data Explored TM software (Applied Biosystems). The relative peak areas were converted to percentages, in which the peak area of corresponding peptides in the control sample was considered as 100%. (**C**) Knockdown of MCP reduced the yield of infectious particles measured by TCID50; (D-E) Following knockdown, the VAS contained deformed intermediates with few viral particles (**D**) or without any viral particles (**E**,**F**) Treatment with the control morpholino shows no negative effect on viral replication; scale bar = 1 μm.

Three peptides of MCP (VSGNPAFGQEFSVGVPR, NLVLPLPFFFSR and SGDYVLNAWLTLK) were detected (Fig. 4A). Following asMO^072^ knockdown, the expression level of MCP was reduced compared to controls (Fig. 4B). The knockdown efficiency as measured by the reduction of different MCP peptides ranged from 50% to 70%, which is likely due to the varying efficiency of detection for the different peptides by MALDI-TOF/TOF MS/MS. The yield of infectious particles in asMO^072^-transfected cells was substantially reduced to 0.1% of that observed in asMO^control^-transfected cells (Fig. 4C). The majority of infected cells with ORF072 knockdown displayed a VAS containing many irregularly shaped, electron dense fiber-like structures but only a few viral particles (Fig. 4D) or no particles (Fig. 4E). In all knockdown cells, no capsid intermediates (two-shelled structures, and crescent or headphone shaped structures) were observed, and the paracrystalline array of virions did not form. In contrast, infected cells transfected with asMO^control^ showed a characteristic VAS with paracrystalline arrays of mature virions (Fig. 4F).

### Budding vacuoles

**Figure 5:**
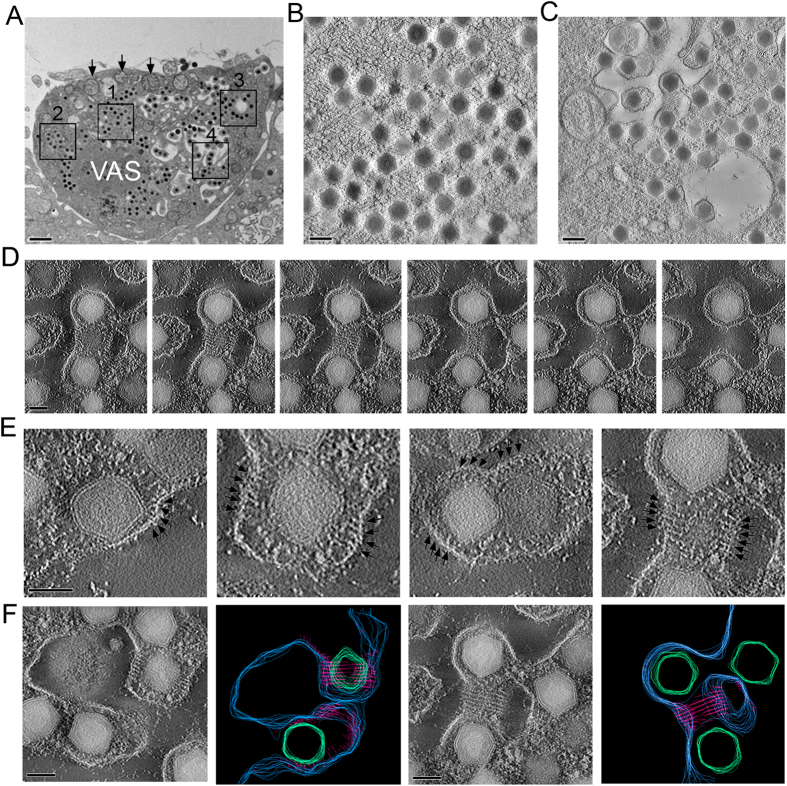
Viral accumulation and egression. (**A**) An electron micrograph of an ultrathin section of SGIV-infected cells (at 8 hpi) reveals the process of intracellular accumulation of viruses and various modes of viral egression including paracrystalline accumulation of mature viral particles (box 1), initiation of vacuolation (box 2), intracellular vacuolation (box 3), accumulation of virus in the form of a tubular vesicle in the vacuole (box 4); scale bar = 1 μm. (**B**) Paracrystalline array of dense viral particles accumulating in the VAS; scale bar = 200 nm. (**C**) Following vacuolation, vesicles containing mature viral particles bud into vacuoles; scale bar = 200 nm. (**D**) A series of slices from an area undergoing membrane tubulation reveals the unique spiral architecture of the membrane-deforming proteins that direct the vacuolar membrane tubulation induced by SGIV infection; scale bar = 100 nm. (**E**) Induction of viral-induced vesicle budding into a tubular structure begins with a recruitment of membrane-bending proteins that bind on the cytosolic side of the vacuolar membrane. The proteins form a unique spiral structure on the membrane, reshaping the vacuolar membrane into a membrane tubule which contains the virus inside the vacuole (arrows), scale bar = 100 nm. (**F**) Segmented contours imaging and the corresponding tomographic sections of viral-induced membrane buds and membrane tubules show the spatial distribution of the unique spiral structures, blue = vesicular membrane; green = viral particles; pink = spiral structures, scale bar = 100 nm.

**Figure 6:**
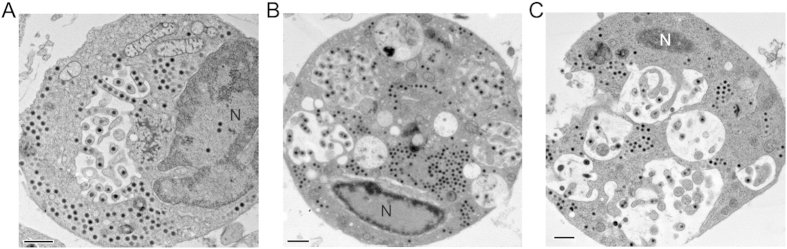
Development of the budding vacuole. (**A**) At the beginning of VAS expansion, the nucleus is still large and few budding vesicles are observed. Some of the mitochondria surrounding the VAS have lost their inner cristae, and may become the vacant vesicle for viral particles to bud into. (**B**) As more vesicles develop and take up space inside the cell, the nucleus reduces in size and is pushed towards the edge of the cell. (**C**) Vesicles fuse with each other to form large vacuoles and eventually create an opening in the cell membrane enabling the release of viral particles. At this stage, the nucleus has dramatically reduced in size and remains at the cell periphery. These images were obtained from SGIV infected GECs at 8 hpi, scale bar = 1 μm.

Tilt series of high pressure freezing, freeze substitution (HPF-FS) specimen were taken to investigate a novel viral egress process in the infected GECs. It is characterized by the formation of a large intracellular vacuole, appears in the regions where the mature virus accumulates, usually very close to the VAS. Notably, some mature viral particles were found to attach and bud into those vacuoles. Figure 5 shows a 3D reconstruction of the budding events that occurred inside the vacuole. Cytosolic membrane-bending proteins were recruited to the vacuole, initiating the budding of the virus into a vacuole (Fig. 5C,D). The buds extended and formed membrane tubules containing a row of viruses inside the vacuole (Fig. 5A). The process of viral-induced vacuolar membrane tubulation appeared to be mediated by cytosolic membrane associated proteins that formed a helical structure around the budding vesicle (Fig. 5D–F). Segmented contour processing was used to further highlight the spiral structures (Fig. 5F). To further determine the role of the intra-vacuolar virus budding in facilitating the dissemination of viral particles, we extensively examined SGIV-infected cells at 4, 8, 24 and 48 hpi. Compared to the process of budding through the cell periphery, more virions were observed in intra-vacuolar membrane tubules, while a vast of majority of virions in paracrystalline arrays are released by cell lysis (Supplementary Fig S2). These budding vacuoles were most prominent at 8 hpi (Fig. 6), with around 30% of total viral particles contained in these vacuolar membrane tubules (Supplementary Table S1). The fusion of individual vacuoles led to the formation of a large vacuole that may subsequently fuse with the cell membrane to create an opening and release the virions out of the cell. In addition, the long tubular structure may extend out from the cell to reach neighboring uninfected cells for direct virion transmission.

## Discussion

During its life cycle, iridovirid produces enveloped and unenveloped viral particles, both of which are infectious. Using the high pressure freezing (HPF) method, which preserves the virus structure much better than conventional chemical fixation^28^, we observed that the unenveloped virions are not naked, but actually have an outer capsid layer (Fig. 1A). Understanding the role of this outermost structure will be important for deciphering the process of viral entry, which has been shown to occur via clathrin-mediated endocytosis or macropinocytosis in a pH-dependent manner^31^. Intriguingly, mild detergent treatment reduces viral infectivity, which can be partially rescued by incubation with lipids from the host cells^28^, suggesting that the outer capsid layer has a key role in promoting infectivity. However, it is clear that the SGIV outermost layer is not a typical envelope. It may be lost after hash purification and was not observed in highly purified viral particles (Fig. 3G,H). An outermost layer has also been observed on lymphocystis disease virus (LCDV), another member of the family Iridoviridae from the genus *Lymphocystivirus*^11^, but with a different morphology.

Viral assembly often takes place in specific intracellular compartments where viral components concentrate, which increases the efficiency of the process^32^. In the case of iridovirids, they assemble within a discrete cytoplasmic area called the viral assembly site (VAS). In SGIV-infected grouper embryonic cells, VAS formation takes place within the first hour of infection. In infected GECs, the VAS is surrounded by mitochondria (Fig. 1B), some of which had lost their internal cristae. Mitochondria are commonly involved in large DNA virus assembly, as seen in the VAS of African swine fever virus (AFSV), which is similarly surrounded by large clusters of mitochondria, although other membrane organelles can be involved, such as rough endoplasmic reticulum, which surrounds the VAS of vaccinia virus^32^. CryoEM also revealed that the VAS has channels connecting it with the nucleus through nuclear pores (Fig. 1C, arrows), and these may facilitate the transport of newly synthesized viral DNA from nucleus to the VAS.

At the early stages of SGIV assembly, we observed a variety of membranous structures which were either linear (membranous sheets) or circular in shape (headphone shaped, crescent shaped intermediates) (Fig. 2A,B), and these structures are likely to arise from ER cisternae. In support of this hypothesis, we have previously demonstrated via lipodomic analysis that SGIV contains a high proportion of phosphatidylinositol, a major component of ER lipids membranes^28^. Similarly, studies of the giant mimivirus has revealed that multi-vesicular bodies rupture to form large, open, single-layered membrane sheets from which viral membranes are generated^33^. Other research also supports the idea that open membranes are precursors for assembly of large DNA viruses^34^.

SGIV lipid-binding proteins, such as ORF026, 075, 089, 090, and 101, may interact with the ER-derived membranes and serve as precursors to recruit capsid proteins to form viral intermediates, crescent-shaped structures with two shells, the inner lipid envelope (black arrow head) and the outer capsid (white arrow head) (Fig. 2C). Envelope proteins identified from other studies, such as *rana grylio* virus (RGV) envelope protein 2L (a homolog of SGIV ORF019)^35^ and FV3 53R (a homolog of SGIV ORF088L)^36^,^37^ may also play a role in the process. When the major capsid protein ORF072 was eliminated by morpholino gene knockdown, the two-shelled crescent structure disappeared (Fig. 4).

The progressive curvature change from crescent shape to hexagonal capsid occurs concurrently with the buildup of electron dense materials underneath the inner lipid envelope (Fig. 2C–F). We assume that some of the lipid binding proteins mentioned above may be involved in the recruitment of this electron dense layer. However, the electron dense materials may not be essential for the curvature change because some hexagonal capsids were able to form without the electron dense materials inside (Fig. 2B). As ASFV has a very similar morphology to iridovirids, the ASFV assembly model is often used to describe iridovirid morphogenesis. The hexagonal capsid of ASFV is formed while the capsid proteins are being gradually assembled to the precursor viral membrane^38^. However, the formation of SGIV hexagonal capsid appears to be different to ASFV with the two-shelled structures forming first, followed by curving into icosahedral particles. This is also different from herpesvirus capsid assembly, in which the capsid undergoes substantial conformational changes from a roughly spherical form to an icosahedral form during the DNA encapsidation and maturation process^39^.

The electron dense materials underneath the inner envelope shell may be involved in viral DNA encapsidation. As the immature particle becomes mature, there is a corresponding increase in density (Fig. 3C–F). Using gold particle labelling we demonstrated that these dense materials are viral DNA. The dense materials filled into the capsid gradually and the capsid gap was sealed with a “sealing mark” on one position (Fig. 3H). We have previously identified 12 proteins associated with the viral core^40^, and two of these SGIV core proteins, ORF018R and ORF008L are involved in viral DNA organization and packaging^41^. Knockdown of the lipid binding protein ORF075, results in the formation of many particles without DNA cores^42^. In other iridovirids, DNA packaging via a headful mechanism has been proposed^43^,^44^. Taken together with our experimental observations, this suggests that viral DNA encapsidation may involve a number of viral proteins, and the detailed mechanism underlying this needs to be explored further.

Most of the viral intermediates were observed in the center of the VAS at 1 or 2 hpi, yet mature virions moved to the VAS periphery, where they either bud out (frequently observed around 6–8 hpi when infected with a MOI of 5–10) or form paracrystalline arrays (often observed after 48 hpi when infected with a MOI of 3 or lower). The budding from the cell periphery has been reported previously^45^, but due to the advantage in sample fixation provided by HPF-FS over conventional chemical fixation, we have uncovered a novel budding process in this study. Intriguingly, budding through this new pathway was observed much more often than budding through the cell periphery.

In the first step of this new budding process, vacuoles were formed around the VAS. It is unknown whether those vacuoles were newly generated or transformed from organelles such as mitochondria. We observed that mitochondria surrounding the VAS were enlarged and lost their internal structures (Figs 1B and 5A, arrowed organelles; Fig. 6). This has also been reported in EPC (Epithelioma papulosum cyprinid) cells following giant salamander iridovirus infection^18^ and in RGV-infected fathead minnow (FHM) cells^46^. Following vacuole formation, the surrounding mature virions budded into the vacuoles in a cluster to form tubular structures. Vacuoles may fuse with neighbor vacuoles to form larger vacuoles, and eventually, an opening in the cell membrane formed, or cell lysis was triggered to release mature virions that are enclosed in cellular membranes (Fig. 6). This novel budding process may reveal a new immune evasion strategy by SGIV. At the early phase of infection, the virions hidden in tubular structures can spread to uninfected neighbor cells with minimal or no exposure to the host immune system. This may also delay the onset of host immune response, such as the production of antiviral interferons. This ability of SGIV to hide in membranous tubular structures helps it to establish an infection and maximizes viral replication. A study of tick-borne encephalitis virus (TBEV) has shown that TBEV infection leads to formation of intracellular membrane vesicles that protect the viral dsRNA from cellular recognition, used as an immune evasion strategy^47^. In addition, there are two infectious vaccinia virus particles, called intracellular mature virus (IMV) and extracellular enveloped virus (EEV). It has been found that EEVs are capable of evading host antibody and complement by wrapping themselves in a host-derived membrane^48^,^49^. At the late phase of SGIV infection, the large number of mature virions in paracrystalline arrays is released by cell lysis to infect other cells. Although there are a few reports describing potent immune evasion mechanisms of ranavirus^50^,^51^,^52^, our research reveals a new mechanism for ranavirus to evade the host immune system.

**Figure 7:**
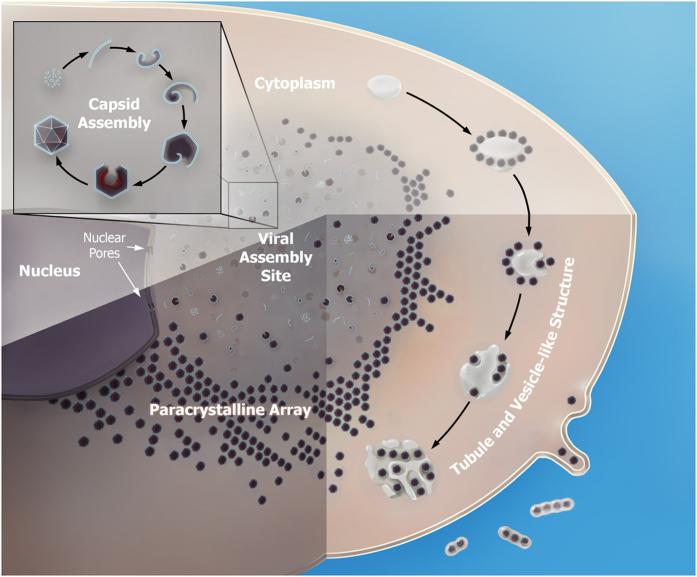
Schematic of the SGIV life cycle. SGIV enters the cell through endocytosis and this leads to the formation of coated vesicles containing virions. These vesicles are transported to the nucleus and release the viral genome into the nucleus. Viral replication results in the formation of the viral assembly site in the cytoplasm, the location for viral morphogenesis. The steps involved in viral morphogenesis and capsid assembly are shown in the inset. Mature virions either accumulate inside the cytoplasm as paracrystalline arrays and are released through host cell lysis, and can also be released by budding, either directly through the cell membrane, or by the formation of budding vacuoles within the cell.

In summary, our study presents the first clear visualization of the outermost layer of unenveloped virions, and we show that this layer is the frontier for viral interaction with the cell membrane for viral entry. Using cryoEM/ET imaging, structural information of viral morphogenesis from early precursors to various intermediates and finally mature particles is also shown for the first time, and formation of mature viral particles requires the major capsid protein ORF072. Taken together, we unravel a detailed life cycle and a novel budding process used by SGIV to disseminate viral particles. The schematic in Fig. 7 summarizes our current understanding of SGIV morphogenesis and life cycle.

## Materials and Methods

### Cell culture, virus infection and purification

Grouper embryonic cells (GECs) from the brown-spotted grouper *Epinephelus tauvina*^53^ were cultured at 27 °C (room temperature, RT) in Eagle’s minimum essential medium containing a final concentration of 10% fetal bovine serum, 162 mM NaCl, 100 IU of penicillin G per ml, 0.1 mg streptomycin sulfate per ml, and 5 mM HEPES. The pH of the medium was adjusted to 7.4 with NaHCO_3_. Freshly confluent monolayers of GECs were infected with SGIV at a multiplicity of infection (MOI) of approximately 1 at RT. When approximately 70% of cells were detached, the medium containing SGIV was harvested and the titers were determined by a TCID_50_ test^30^. Viruses were purified by discontinuous sucrose gradients followed by cesium chloride gradients^40^.

### EM sample preparation

GECs were infected with SGIV at different MOI values and temperatures. Infection with high MOIs (more than 10) at 4 °C was carried out to examine events at early stages of the viral life cycle (0.5, 1 and 2 hours post-infection[hpi]), while infection at low MOIs at 27 °C allowed the capture of events at late stages of the viral life cycle (2, 8, 24 and 48 hpi). The infected cells were harvested by centrifugation at different time points. Infected GECs were then subjected to high pressure freezing (HPF, cryofixation), freeze substitution (FS), ultramicrotomy, and staining, before observation under a FEI Tecnai T12 electron microscope.

### Cryofixation, freeze substitution, ultramicrotomy and staining

Cryofixation of SGIV-infected cells was performed using an HPF Compact 01 system. In brief, about 3 μl of sample was inserted in a hexadicine coated aluminum platelet, quickly frozen by the HPF machine, and then transferred to vials containing 1% osmium tetroxide (OsO4), 0.5% uranyl acetate, and 5% H_2_O in acetone for freeze substitution (FS), which was carried out using a Leica FS machine, followed by epoxy infiltration as per the previously described protocol^54^. Ultrathin sections (7090 nm) were cut using a Leica ultramicrotome UCT, placed on carbon-coated grids and double stained with 2% uranyl acetate and Reynold’s lead citrate^55^.

### Electron Tomography (ET) and 3D reconstruction

The specimens were visualized on the FEI Tecnai T12 electron microscope operated at 120kv. The tilt series were recorded using the FEI tomography software from –60° to +60° with 2° intervals at a defocus of 6 μm on a 4k x 4k CCD camera (Gatan), with magnification was set to 5,000–25,000x depending on the size of the viral assembly intermediates. The tilt series were aligned using IMOD software package^56^ and a final 3D reconstruction was computed using the simultaneous iterative reconstruction technique (SIRT) algorithm. For all segmented-tomograms, a Gaussian filter was applied to reduce noise and improve contrast of the tomograms before they were subjected to semi-automated threshold segmentation. Images were rendered using the Chimera software package^57^.

### Quantification of capsid intermediates

GEC cells infected with SGIV were maintained at 4 °C and 27 °C after infection and collected at 1 and 2 hpi. Based on the classification of capsid intermediates (Supplementary Fig S1), the number of each intermediate was counted from two batches of cryoEM samples at each temperature and time point.

### CryoEM of SGIV particles

Cryofixation of SGIV particles was done by a plunge freezer (Vitrobot Mark IV). Approximately 3μl of purified virus particles was applied onto a glow discharged quantifoil grid with a hole size of 2 μm. The grid was plunge-frozen in liquid ethane, blotted for 1 second at 100% humidity. The specimen was visualized using a Titan Krios electron microscope with the defocus ranging from −1.5 to −3μm, at a dose of 16 to 20e/Å^2^. Images were recorded using a Gatan Ultrascan 4k × 4k CCD detector with magnification of 63,290×.

### Gene knockdown by antisense Morpholino oligonucleotide (asMO)

The asMO sequences of SGIV major capsid protein ORF072 (abbreviated to asMO^072^) and control (asMO^control^) are 5′-CGCCAGCACCCGTTGTACAAGTCAT-3′ and 5′-CCTCTTACCTCAGTTACAATTTATA-3′, respectively. These sequences were designed to bind the target mRNA around the start codon region and block translation. The asMO was transfected into GECs by electroporation via a nucleofector machine as previously described^58^. Briefly, fresh confluent GECs were transfected with 20μM of asMO^072^ and asMO^control^, incubated for 40 h, followed by inoculation with SGIV at an MOI of 3, and incubated for another 48 h before harvesting for subsequent analysis.

Reduction of MCP (ORF072): The efficiency of knockdown in the reduction of MCP expression was assessed by label-free quantitative proteomics^59^,^60^. Briefly, the total amount of protein from the cell lysates of both asMO^072^ and asMO^control^ groups were quantified by RC DC protein assay from Bio-Rad, and separated by 1D SDS-PAGE. The protein bands which correlated to the mass of MCP were sliced out and analyzed by matrix assisted laser desorption/ionization (MALDI-TOF/TOF MS/MS). The peak areas of peptides corresponding to MCP were calculated using Data Explorer TM Software (Applied Biosystems), with a large peak area representing a high amount of the protein. The relative amount of MCP was shown as the percentage of the peak area in the knockdown samples divided by the corresponding peak area in the control samples. The lower the percentage, the more efficient the knockdown.

Formation of viral particles: Following transfection, harvested cells were processed for cryoEM to visualize the effect of MCP knockdown on virus assembly. The knockdown effects on the yield of infectious particles were quantitatively assessed using TCID_50_^58^. Briefly, SGIV infected knockdown cells were freeze thawed, and then lysed by multiple passes (5–10) through a 20-gauge syringe needle. After centrifugation of the lysate spins at 1,000 x *g* for 1 min, the supernatant was collected and underwent serial dilution for final solutions of 10^2^ to 10^10^ for TCID_50_ test in GECs.

### Cryo-sectioning and immunolabeling

GECs infected with SGIV at 27 °C for 24 h were processed using Tokuyasu method^61^,^62^ with modifications. Briefly, the infected cells were fixed with 2% paraformaldehyde, 0.2% glutaraldehyde in PBS (pH 7.4) for at least 2h at RT, embedded in 12% wt/vol gelatin and infiltrated with 2.3 M sucrose overnight before cryoultramicrotomy. Ribbons of ultrathin sections (~90 nm) were sectioned at −80 °C using a cryoimmuno diamond knife on a Leica UCT ultramicrotome. Cryo sections were retrieved and thawed to 4 °C by adding a drop of an ice-cold solution, which consisted of 2 parts 2.3M sucrose: 1 part 2% methycellulose.

For immunolabeling, ultrathin sections were first incubated in 0.12% Glycerin for 15 min, followed by blocking with 1% BSA for 30 mins and then washing three times with PBS. The primary antibody of anti-SGIV core-associated protein ORF075 was generated as a gift from the National Institute of Diagnostics and Vaccine Development for Infectious Diseases in Xiamen University, Xiamen, China, following the approved institutional protocol. Mouse anti-DNA antibody was purchased from Millipore (MAB1293). The two antibodies were added at a dilution of 1:50 and incubated for 1 h, washed three times with PBS, followed by conjugation with goat anti-rabbit gold particles (25nm diameter, for labeling ORF075) and the goat anti-mouse gold particles (15 nm diameter, for labeling DNA, Electron Microscopy Sciences, EMS-25132) at a dilution of 1:40 for 1h. Grids were fixed with 1% glutaraldehyde for 5 min, washed four times with water, and incubated with a mixture of 1 part of 1% uranyl acetate: 9 parts of 2% methylcellulose for 5 min. Grids were dragged on a filter paper to remove the excess liquid and air dried before viewing using a FEI Tecnai T12 microscope.

## Additional Information

**How to cite this article**: Liu, Y. *et al.* Visualization of Assembly Intermediates and Budding Vacuoles of Singapore Grouper Iridovirus in Grouper Embryonic Cells. *Sci. Rep.* **6**, 18696; doi: 10.1038/srep18696 (2016).

## Supplementary Material

Supplementary Information
