# Supplementary material for: Visualization of Assembly Intermediates and Budding Vacuoles of Singapore Grouper Iridovirus in Grouper Embryonic Cells
Source: Sci Rep. 2016 Jan 4;6:18696. doi: 10.1038/srep18696 (PMC4698634; doi:10.1038/srep18696)
Supplement: Supplementary Information [file srep18696-s1.doc]

**Visualization of Assembly Intermediates and Budding Vacuoles of Singapore Grouper Iridovirus in Grouper Embryonic Cells**

Yang Liu^1*^, Bich Ngoc Tran^2*^, Fan Wang^1^, Puey Ounjai^3^, Jinlu Wu^1**^ and Choy L. Hew^1,2**^

1. Mechanobiology Institute, National University of Singapore, Singapore 114543

2. Department of Biological Sciences; National University of Singapore, Singapore 114543

3. Department of Biology, Faculty of Science, Mahidol University, 272 Rama VI Rd. Rajdevi, Bangkok, Thailand 10400

*Co-first author;

**Co-corresponding author

Dr. Jinlu Wu

[dbswjl@nus.edu.sg](mailto:dbswjl@nus.edu.sg)

Tel: 65-65168476

Prof Choy L. Hew

[dbshewcl@nus.edu.sg](mailto:dbshewcl@nus.edu.sg)

Tel: 65-65167658

Supplementary Figure S1. The representatives of viral intermediates


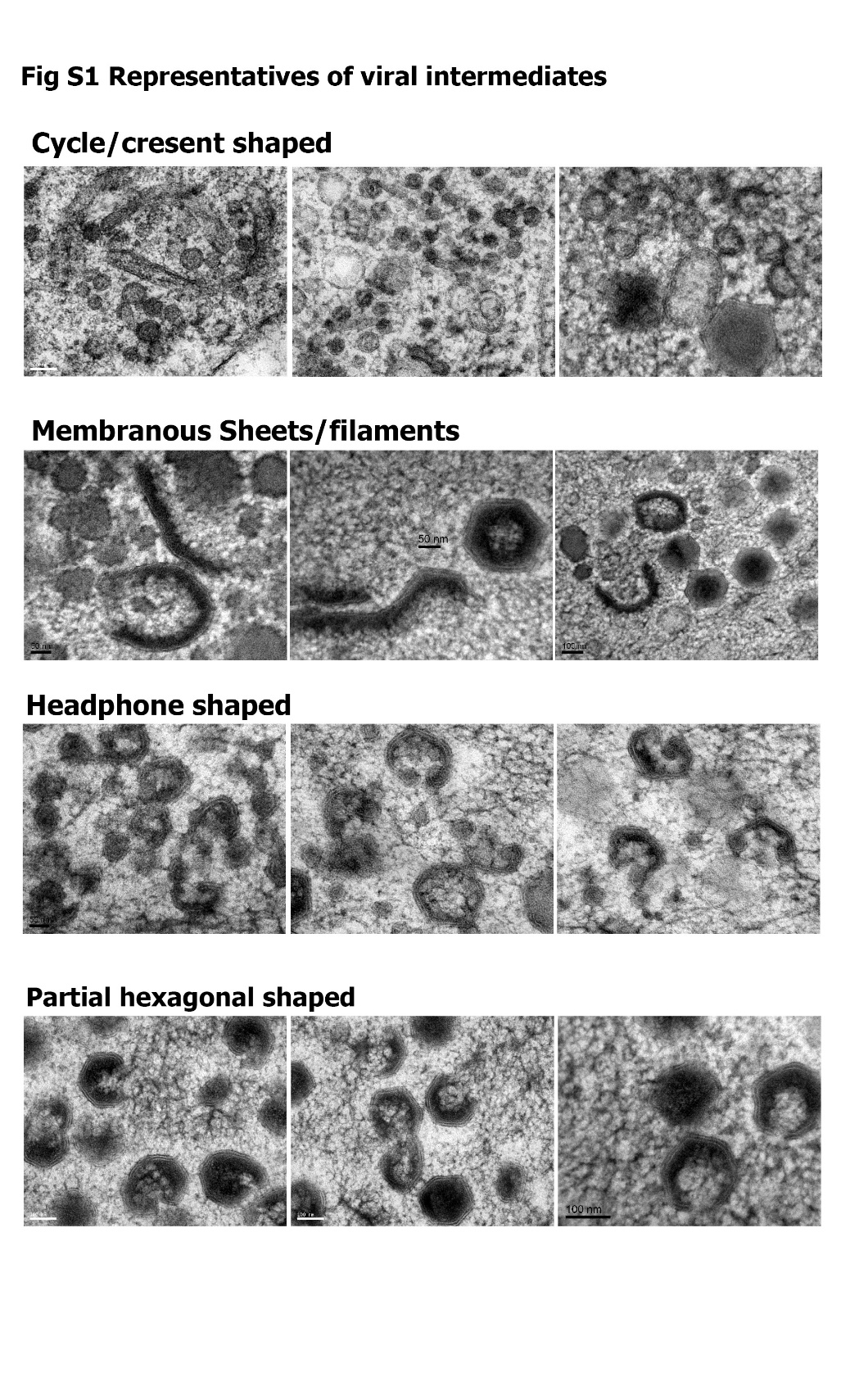


Supplementary Figure S2. Three pathways of SGIV egress: budding through cell periphery (solid black arrow on image A), budding through vacuoles (empty black arrows, image B) and cell lysis for release of viruses in paracrystalline (arrow head, image B). According to all images observed, we estimate that budding through of cell periphery releases the least virions, while lysis releases the most. The progeny virions produced through budding have envelopes, while progeny virions released through lysis may not have envelopes. The enveloped and unenveloped virions may be released at different stages of viral infection, which may serve different purposes for the viral spread and infection.

A. B.


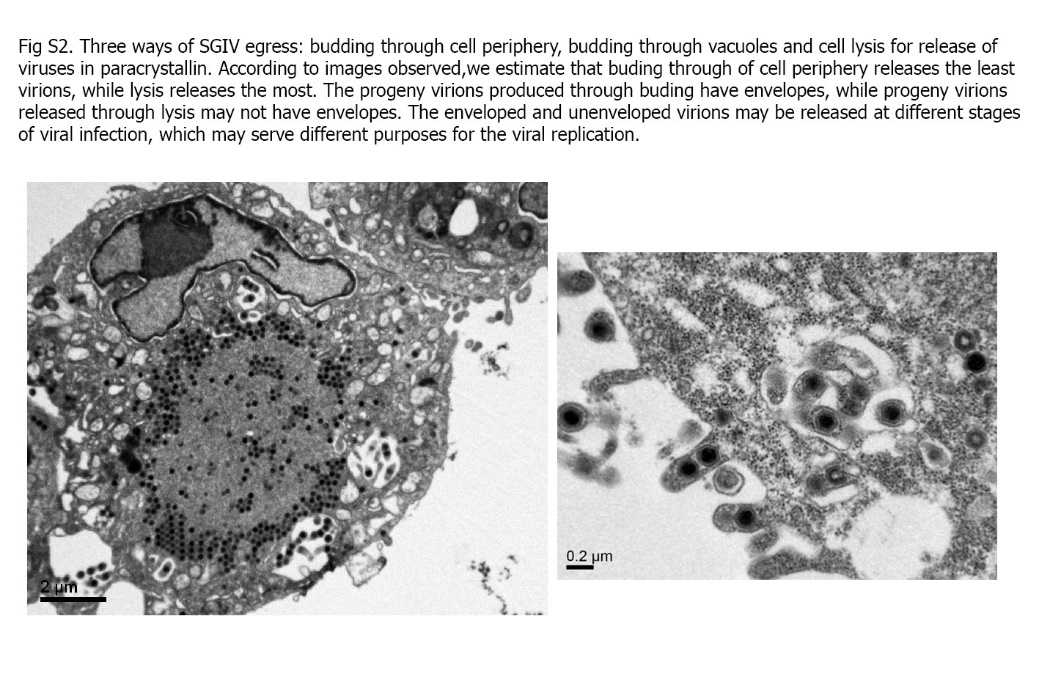

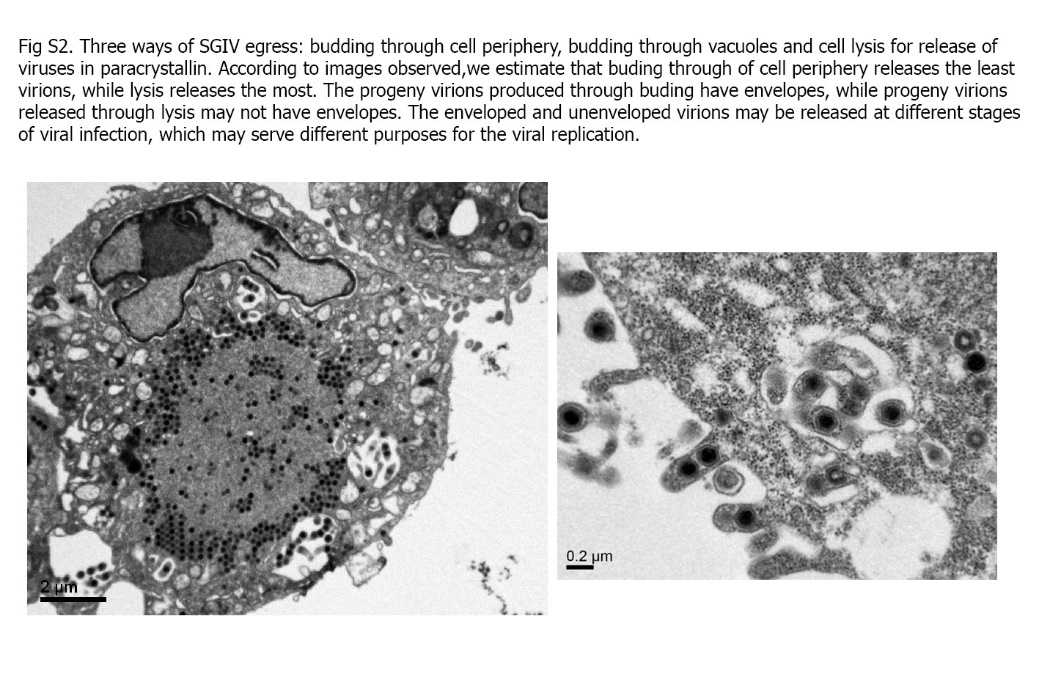


Supplementary Table S1. The number and percentage of virions in the budding vacuoles. The cell sections were from samples collected at 8 hours post-infection.

| Cell section | Number of viruses in the vacuole | Total number of viruses | Percentage |
| --- | --- | --- | --- |
| Section 1 | 48 | 153 | 31.3% |
| Section 2 | 46 | 131 | 35.1% |
| Section 3 | 56 | 175 | 32% |
| Section 4 | 39 | 144 | 27.1% |
| Section 5 | 77 | 276 | 27.9% |
| Total | 266 | 879 | 30.3% |
